# Supplementary material for: Dopamine Therapy and the Regulation of Oxidative Stress and Mitochondrial DNA Copy Number in Patients with Parkinson’s Disease
Source: Antioxidants (Basel). 2020 Nov 20;9(11):1159. doi: 10.3390/antiox9111159 (PMC7699910; doi:10.3390/antiox9111159)
Supplement: Supplementary file 1 [file antioxidants-09-01159-s001.pdf]

Supplemental Table 1. **A** bivariate correlative study identifies some relationships between these identified PD-related biomarkers

		Parkinson dis.	Age	Sex	BMI	TBARS	Thiols	Log_ΔCt copy no
Parkinson dis.	Pearson Correlation	1	.042	.005	-.087**	.163**	-.093**	-.160**
	Sig. (2-tailed)		.109	.861	.005	.000	.000	.000
	N	1469	1468	1469	1032	1430	1469	1448
Age	Pearson Correlation	.042	1	-.075**	-.099**	.042	-.306**	-.127**
	Sig. (2-tailed)	.109		.004	.001	.112	.000	.000
	N	1468	1468	1468	1032	1429	1468	1447
Sex	Pearson Correlation	.005	-.075**	1	-.010	.051	.045	-.056*
	Sig. (2-tailed)	.861	.004		.757	.053	.087	.032
	N	1469	1468	1469	1032	1430	1469	1448
BMI	Pearson Correlation	-.087**	-.099**	-.010	1	.042	.011	.000
	Sig. (2-tailed)	.005	.001	.757		.184	.718	.995
	N	1032	1032	1032	1032	1008	1032	1020
TBARS	Pearson Correlation	.163**	.042	.051	.042	1	-.082**	-.047
	Sig. (2-tailed)	.000	.112	.053	.184		.002	.080
	N	1430	1429	1430	1008	1430	1430	1409
Thiols	Pearson Correlation	-.093**	-.306**	.045	.011	-.082**	1	.063*
	Sig. (2-tailed)	.000	.000	.087	.718	.002		.016
	N	1469	1468	1469	1032	1430	1469	1448
Log_ΔCt copy no	Pearson Correlation	-.160**	-.127**	-.056*	.000	-.047	.063*	1
	Sig. (2-tailed)	.000	.000	.032	.995	.080	.016	
	N	1448	1447	1448	1020	1409	1448	1448

**. Correlation is significant at the 0.01 level (2-tailed).

*. Correlation is significant at the 0.05 level (2-tailed).
